# Mapping of Genetic Abnormalities of Primary Tumours from Metastatic CRC by High-Resolution SNP Arrays

**DOI:** 10.1371/journal.pone.0013752

**Published:** 2010-10-29

**Authors:** José María Sayagués, Celia Fontanillo, María del Mar Abad, María González-González, María Eugenia Sarasquete, Maria del Carmen Chillon, Eva Garcia, Oscar Bengoechea, Emilio Fonseca, Marcos Gonzalez-Diaz, Javier De Las Rivas, Luís Muñoz-Bellvis, Alberto Orfao

**Affiliations:** 1 Servicio General de Citometría, Departamento de Medicina and Centro de Investigación del Cáncer (IBMCC-CSIC/USAL), Universidad de Salamanca, Salamanca, Spain; 2 Grupo de Investigación en Bioinformática y Genómica Funcional, Centro de Investigación del Cáncer (IBMCC-CSIC/USAL), Universidad de Salamanca, Salamanca, Spain; 3 Departamento de Patología, Hospital Universitario de Salamanca, Salamanca, Spain; 4 Servicio de Hematología, Hospital Universitario, Centro de Investigación del Cáncer (IBMCC-CSIC/USAL), Salamanca, Spain; 5 Unidad de Genómica y Proteómica, Centro de Investigación del Cáncer (IBMCC-CSIC/USAL), Universidad de Salamanca, Salamanca, Spain; 6 Servicio de Oncología Médica, Departamento de Cirugía, Hospital Universitario de Salamanca, Salamanca, Spain; 7 Unidad de Cirugía Hepatobiliopancreática, Departamento de Cirugía, Hospital Universitario de Salamanca, Salamanca, Spain; The University of Hong Kong, Hong Kong

## Abstract

**Background:**

For years, the genetics of metastatic colorectal cancer (CRC) have been studied using a variety of techniques. However, most of the approaches employed so far have a relatively limited resolution which hampers detailed characterization of the common recurrent chromosomal breakpoints as well as the identification of small regions carrying genetic changes and the genes involved in them.

**Methodology/Principal Findings:**

Here we applied 500K SNP arrays to map the most common chromosomal lesions present at diagnosis in a series of 23 primary tumours from sporadic CRC patients who had developed liver metastasis. Overall our results confirm that the genetic profile of metastatic CRC is defined by imbalanced gains of chromosomes 7, 8q, 11q, 13q, 20q and X together with losses of the 1p, 8p, 17p and 18q chromosome regions. In addition, SNP-array studies allowed the identification of small (<1.3 Mb) and extensive/large (>1.5 Mb) altered DNA sequences, many of which contain cancer genes known to be involved in CRC and the metastatic process. Detailed characterization of the breakpoint regions for the altered chromosomes showed four recurrent breakpoints at chromosomes 1p12, 8p12, 17p11.2 and 20p12.1; interestingly, the most frequently observed recurrent chromosomal breakpoint was localized at 17p11.2 and systematically targeted the *FAM27L* gene, whose role in CRC deserves further investigations.

**Conclusions/Significance:**

In summary, in the present study we provide a detailed map of the genetic abnormalities of primary tumours from metastatic CRC patients, which confirm and extend on previous observations as regards the identification of genes potentially involved in development of CRC and the metastatic process.

## Introduction

The development and progression of CRC is a multistep process leading to the accumulation of genomic alterations that occur at the single cell level over the lifetime of a tumour, from benign to invasive and metastatic states leading to patient death [1], [2]. For many years, the genetics of metastatic CRC have been studied with an increasingly high variety of techniques from conventional cytogenetics [3] and fluorescence *in situ* hybridization (FISH) [4] to comparative genomic hybridization (CGH) [5] and array CGH (aCGH) [6]. Based on these techniques, many different recurrent genetic abnormalities have been identified in metastatic CRC which frequently include gains of chromosomes 8q, 13q and 20q [7], [8] together with losses of the 1p, 8p, 17p and 18q chromosomal regions [9]. By contrast, detailed characterization of the common breakpoint regions as well as the identification of the specific genes targeted by such abnormalities has proven difficult with these approaches. This is partially due to the fact that these techniques have a relatively limited resolution which hampers identification of the specific cancer-associated genes recurrently targeted in such alterations. In fact, the highest resolution approaches applied so far to the study of CRC are based on aCGH (i.e. Camps *et al* who applied a 185K oligonucleotide array with an estimated resolution of 16 kb, to the analysis of 32 primary CRC tumours) [10].

In recent years, the availability of high-density single nucleotide polymorphism (SNP) arrays has allowed identification of small regions of chromosomal gains and losses with a much higher resolution, down to 2.5 kb [11]. Thus, based on genome wide SNP arrays, fine mapping of chromosomal breakpoints and subsequent identification of the specific genes recurrently altered (deleted, gained or amplified) is achieved for individual samples. This allows for a more precise and detailed comparison of the breakpoint regions found in different tumours and their correlation with the clinical features of the disease.

In the present study we used 500K SNP mapping arrays with a mean distance between interrogated SNPs of 5.8 kb (median intermarker distance of 2.5 kb) to map genetic lesions present at diagnosis in primary tumours from a group of 23 sporadic CRC patients who developed liver metastasis. Our major goal was to define the most frequent recurrent breakpoint regions in metastatic CRC and the commonly gained and/or deleted genes in the altered chromosomes. In order to evaluate the reproducibility of the SNP-array results we performed parallel interphase FISH (iFISH) analyses of the same tumour samples using 24 probes directed against an identical number of regions from 20 different human chromosomes frequently altered in sporadic CRC.

## Materials and Methods

### Patients and samples

Tissue specimens were obtained from primary tumours from 23 patients (15 males and 8 females; median age of 68 years, ranging from 48 to 80 years) suffering from metastatic sporadic CRC. The study was approved by the local ethics committee of the University Hospital of Salamanca (Salamanca, Spain) and prior to entering to the study, informed consent was given by each individual.

In each case, the diagnosis and the classification of the tumours were performed according to the WHO criteria [12]. According to tumour grade, 13 cases corresponded to well-differentiated CRC, 8 to moderately- and 2 to poorly-differentiated tumours. Histopathological grade was confirmed in all cases in a second independent evaluation by an experienced pathologist.

From the 23 primary tumors, 16 were localized at the right (caecum, ascending or trasverse) or the left (descending and sigmoid) colon and 7 in the rectum. Mean size of primary tumors was of 5.2±1.8 cm with the following distribution according to the TNM stage [13]: T3N0M1, 3 cases; T3N1M1, 9; T3N2M1, 3; T4N0M1, 5; T4N1M1, 1 and; T4N2M1, 2 patients. In all cases paired liver metastases were identified either at the time of colorectal surgery (n = 14) or during the first year after initial diagnosis (n = 9); the mean size of the largest liver metastases/patient was of 5.3±2.8 cm (range: 2 to 10 cm).

After histopathological diagnosis was established, samples from representative areas of the primary tumours showing macroscopical infiltration, were used to prepare single cell suspensions to be stored (−20°C) in methanol/acetic (3/1; vol/vol) for further iFISH analyses [14]. The remaining tissue was either fixed in formalin and embedded in paraffin or frozen in liquid nitrogen, and stored at room temperature (RT) and at −80°C, respectively. From the paraffin-embedded tissue samples, sections were cut from three different areas representative of the tumoural tissue used to prepare single cell suspensions and placed over poly L-lysine coated slides. All tissues were evaluated after hematoxylin-eosin staining to confirm the presence of tumour cells and evaluate their quantity in samples to be studied by both iFISH and SNP-arrays. For SNP-array studies, tumour DNA was extracted from freshly-frozen tumour tissues mirror cut to those used for iFISH analyses which contained ≥65% epithelial tumour cells. In turn, normal DNA was extracted from matched peripheral blood (PB) leucocytes from the same patient. For both types of samples (tumour tissue and PB leucocytes), DNA was extracted using the QIAamp DNA mini kit (Qiagen, Hilden, Germany) following the manufacturer's instructions.

### Analysis of single nucleotide polymorphism (SNP) arrays

Paired samples of purified tumoural DNA and normal PB DNA from individual patients were hybridized to two 250K Affymetrix SNP Mapping arrays (*Nsp*I and *Sty*I SNP arrays, Affymetrix, Santa Clara, CA) using a total of 250 ng of DNA per array, according to the instructions of the manufacturer. Fluorescence signals were detected using the GeneChip Scanner 3000 (Affymetrix). Average genotyping call rates of 94.4% and 97.3% were obtained for tumoral and paired normal PB DNA samples, respectively. Only those SNPs with a call rate ≥92.3% were used for further analyses.

In order to calculate genome-wide copy number (CN) changes in tumoural vs. normal samples, the *aroma.affymetrix* algorithm was used, following the CRMA v2 method, as described elsewhere (R-software package, Berkeley, CA) [15]. The following sequential steps were used for this purpose: i) calibration for crosstalks between pairs of allele probes; ii) normalization for probe nucleotide-sequence effects, and; iii) normalization for PCR fragment length- and probe localization-dependent effects. Then, data derived from both the 250K *Sty*I and the 250K *Nsp*I arrays was integrated into a single database and raw CN values calculated as transformed log2 values of the tumoural/normal ratio obtained for paired SNP fluorescence signals.

Log2 ratio values were then used to identify DNA regions which showed similar CN values, using the Circular Binary Segmentation (CBS) algorithm [16]. For the identification of altered (gained or lost) DNA regions, a threshold was established based on the changes observed in the log2 CN values (fluorescence intensity ratio) of sequential tumour DNA segments found for each individual. Therefore, log2 ratio >0.09 and <−0.09 were used as cut-off thresholds to define the presence of increased and decreased CN values, respectively. High-level gains (amplifications) were defined as regions with a mean log2 CN ratio ≥0.22 for ≥3 contiguous SNPs. The specific frequencies of both CN gains and losses per SNP were established and plotted along individual chromosomes for each individual case analyzed. Minimal common regions (MCR) of gain and loss were defined as the smallest group of contiguous SNPs (≥3) with a high frequency of gains and losses (Z-score threshold ≥2.1) according to the overall distribution of CN values found in the entire tumour cell genome, respectively. Common recurrent breakpoint regions were defined as those chromosomal regions which recurrently showed transition from one CN state (gain, loss or no-change) to another for the whole set of individual samples analyzed, at a frequency of ≥35% of the cases (n = 8/23 samples).

### Interphase fluorescence in situ hybridization (iFISH) studies

In all cases, iFISH studies were performed on an aliquot of the single cell suspension prepared from the tumour sample. A set of 24 locus-specific FISH probes directed against DNA sequences localized in 20 different human chromosomes, specific for those chromosomal regions more frequently gained or deleted in sporadic CRC [4], [6], [8], [17], [18] were systematically used to validate the results obtained with the SNP arrays (Table 1).

**Table 1:** A panel of 24 locus-specific FISH probes directed against 24 different regions localized in 20 different human chromosomes were used to validate the results obtained with the SNP arrays.

iFISH probe chromosome localization	iFISH probe length (kb)	Target gene	N. of SNPs inside the region identified by the iFISH probe
1p36	110	*P58*	120
1q25	620	*ABL2*	68
2p24	200	*NMYC*	38
3q26	839	*HTERC*	52
5p15.2	450	*D5S721*	118
6q23	740	*MYB*	88
7q31	200	*D7S486*	33
8p22	170	*LPL*	39
8q24	600	*CMYC*	159
9p21	190	*P16*	37
9q34	270	*ABL1*	33
10q23	370	*PTEN*	49
11q22	184	*ATM*	69
12p13	350	*TEL*	98
13q14	220	*RB1*	14
13q34	550	*LAMP1*	92
14q32	1500	*IGH*	82
15q22	540	*DAPK2*	38
17p13	145	*TP53*	12
18q21	750	*BCL2*	153
19q13	340	*CD37*	21
20q13.2	320	*ZNF217*	53
21q22	500	*AML1*	111
22q11.2	300	*BCR*	36

All probes were purchased from Vysis Inc (Chicago, IL, USA), except for the 3q26, 15p22 and 19q13 probes, which were obtained from QBIOgene Inc (Amsterdam, The Netherlands).

The methods and procedures used for the iFISH studies have been previously described in detail [19]. Briefly, dried slides containing both the tumour cells' and the probes' DNA were denatured (1 min at 75°C) and hybridized overnight (37°C) in a Hybrite termocycler (Vysis Inc, Downers Grove, IL, USA). After this incubation, slides were sequentially washed (5 min at 46°C) in 50% formamide in a 2× saline sodium citrate buffer (SSC) and in 2XSSC. Finally, nuclei were counterstained with 35 μL of a mounting medium containing 75 ng/ml of 4,6-diamidino 2-phenylindole (DAPI; Sigma, St Louis, MO, USA); Vectashield (Vector Laboratories Inc, Burlingame, CA, USA) was used as antifading agent.

A BX60 fluorescence microscope (Olympus, Hamburg, Germany) equipped with a 100× oil objective was used to count the number of hybridization spots/nuclei for ≥200 cells/sample. Only those spots with a similar size, intensity and shape were counted in areas with <1% unhybridized cells; doublet signals were considered as single spots. A tumour was considered to carry a numerical abnormality for a given chromosomal region when the proportion of cells displaying an abnormal number of hybridization spots for the corresponding probe was at a percentage higher or lower than the mean value plus two standard deviations (SD) of the mean percentage obtained with the same probe in control samples (n = 10).

### Quantitative Real-Time PCR

In order to validate the results obtained in the SNP-array studies, quantitative real-time polymerase chain reaction (RQ-PCR) was performed using the Step One Plus Real-Time PCR System (Applied Biosystems, Foster City, CA) in matched normal and tumoural samples in 18/23 cases. Expression of the *MAP2K4*, *MYC* and *BIRC7* genes was analyzed. We employed TaqMan® Gene Expression Assays designed by Applied Biosystems (Applied Biosystems, Foster City, CA) according to the manufactureŕs instructions, and the assays ID for the genes studied were as follows: Hs_00387426-m1 (*MAP2K4*), Hs_00153408-m1 (*MYC*) and Hs_00223384-m1 (*BIRC7*).

Each PCR was carried out in duplicate in a 10 uL volume using the TaqMan® Fast Universal Mastermix (Applied Biosystems) and the following cycling parameters: incubation at 95°C (20 sec), followed by 50 cycles at 95°C (1 sec) and an incubation at 60°C (20 sec). Analysis was made using StepOne software v2.0. The obtained data were normalized by using the internal housekeeping gene, *GAPDH*. Relative quantification was calculated using the equation 2^−ΔCT^ =  C_TGENE_-C_T*GAPDH*._ The final mRNA expression index in each sample was calculated as follows (arbitrary units; AU): mRNA expression index  =  *MYC* or *MAP2K4* or *BIRC7* mRNA value/ *GAPDH* mRNA value X 10,000 AU.

### Statistical methods

For all continuous variables, mean values (and SD) and range were calculated using the SPSS software package (SPSS 12.0 Inc, Chicago, IL USA); for dichotomic variables, frequencies were reported. In order to evaluate the statistical significance of differences observed between groups, the Mann-Whitney U and X^2^ tests were used for continuous and categorical variables, respectively (SPSS).

A multivariate stepwise regression analysis (regression, SPSS) was performed to determine the correlation between the structural and/or numerical abnormalities found for both iFISH, SNP-array techniques and their relationship with the expression of those genes analyzed by RQ-PCR. Only those iFISH probes with ≥12 SNPs localized in the iFISH mapped region (Table 1) were used for correlation studies with the CN status identified by the SNP array (gain vs. loss vs. no change) for those SNPs localized at each iFISH region. *P*-values <.01 were considered to be associated with statistical significance.

## Results

### Map of CN changes by SNP arrays

**Figure 1:**
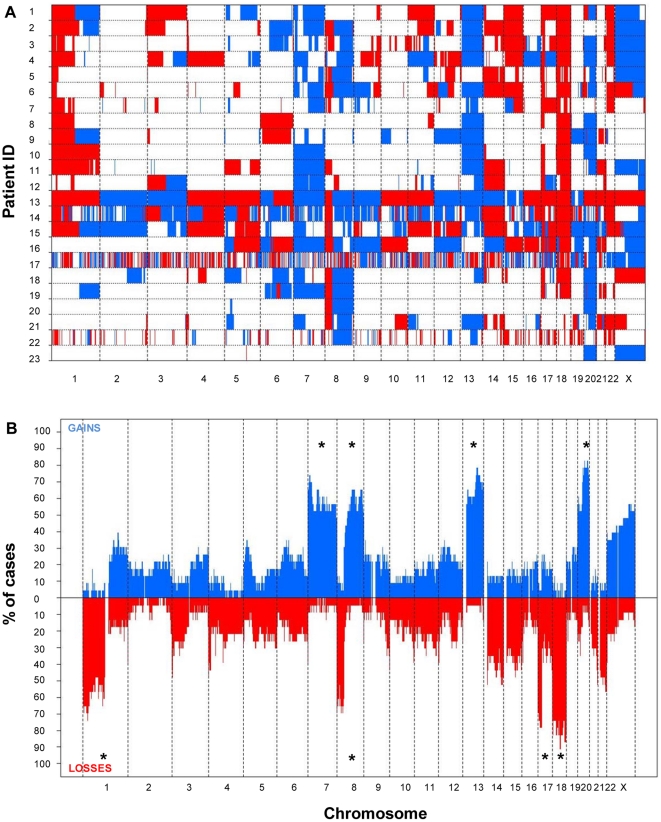
Metastatic colorectal cancer genome for the 23 CRC patients studied. In panel A an overall view of both the gained (blue areas) and lost (red areas) chromosome regions across the genome are shown for the 23 patients genotyped on the Affymetrix 500k SNP array platform. In panel B a summary plot showing the frequency of CN gains (plotted above zero values in the x-axis) and losses (plotted below zero values the x-axis) detected for each individual chromosome, is displayed. Those chromosome regions most frequently showing recurrent losses and gains by SNP arrays were localized in chromosomes 1p, 8p, 17p and 18, and involved the whole chromosome 7 and the 8q, 13q and 20q chromosome regions, respectively.

**Figure 2:**
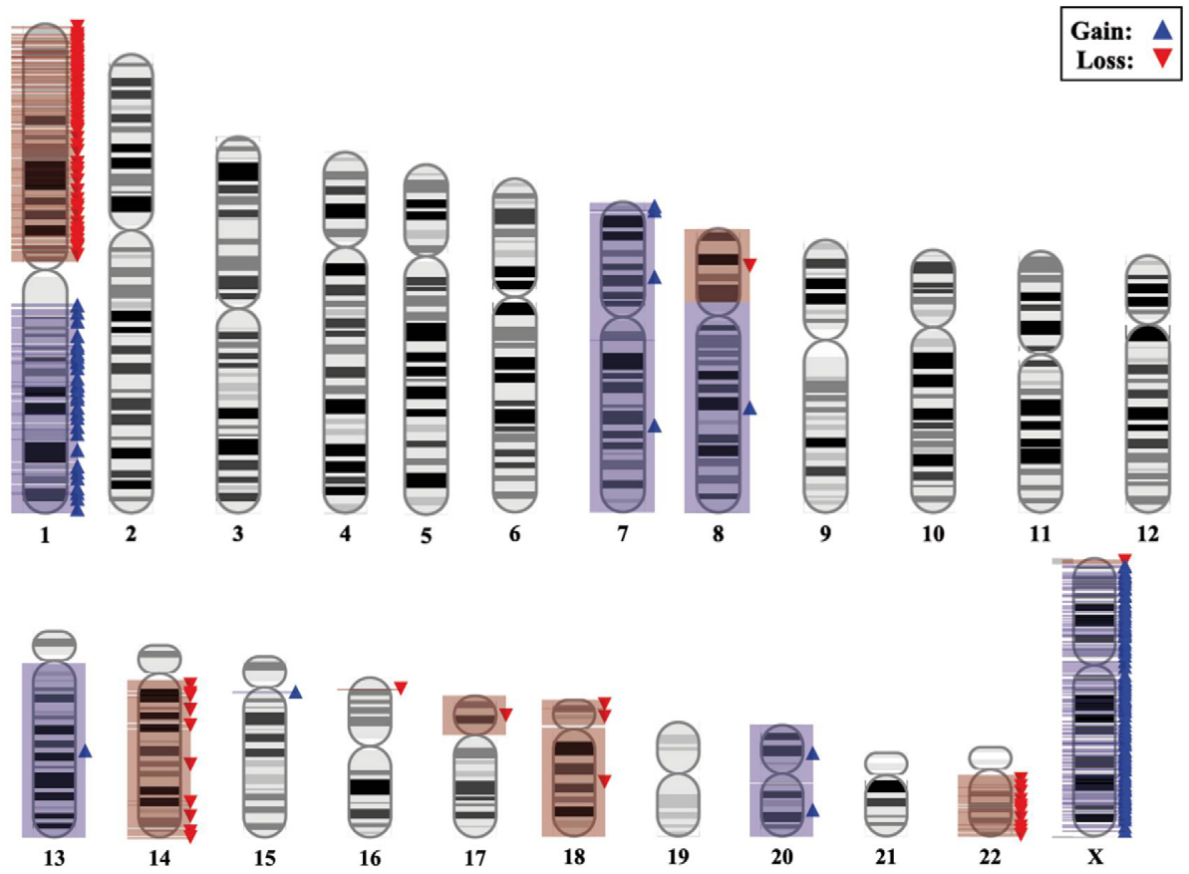
Representative karyotype of a primary metastatic colorectal tumor as determined by the Affymetrix 500K SNP array genotyping platform, showing summary results for those chromosome gains/losses more frequently detected in the colorectal tumor samples analyzed (n = 23).

Overall CN changes for at least one chromosomal region were detected in all 23 tumors studied. The highest frequency of CN losses detected corresponded to chromosomes 1p (n = 17; 74%), 8p (n = 18; 78%), 14q (n = 15; 65%), 17p (n = 19; 83%), 18 (n = 21; 91%) and 22q (n = 17; 74%); in turn, CN gains more frequently involved chromosomes 1q (n =  10; 43%), 7 (n = 20; 87%), 8q (n = 17; 74%), 13q (n = 18; 78%), 20q (n = 20; 87%) and X (n = 13; 57%) (Figure 1); these (gained) chromosomes/chromosomal regions also revealed the highest level of genomic amplification (Table S1). In addition, gains and losses of many other chromosomal regions were identified at lower frequencies (Figure 1). An illustrating map of the most frequently gained/lost chromosome regions according to SNP-array studies, is shown in figure 2.

Of note, SNP arrays allowed the identification of 43 small DNA sequences (arbitrarily defined as regions of <1300 kb) which displayed recurrent CN changes (gains and losses). Interestingly, most of those regions which showed recurrent CN changes (n = 28/43) contained at least one known well-characterized gene, five contained known cancer-associated genes and one region held a microRNA gene (*MIR1208*), localized at chromosome 8q24.21 (Table 2). The exact number of small regions characterized by CN changes, as well as the relative proportion of CN gains vs. losses varied widely among the different chromosomes. The 43 small regions containing CN gains and losses were coded in those chromosomes more frequently affected by CN changes and their distribution was as follows: chromosomes 1p, 1 region; 7p, 3; 8p, 4; 8q, 16; 13q, 7; 17p, 3; 18q, 4; 20q, 3, and; Xq, 2 region. In addition, other regions carrying recurrent large-scale CN gains and losses (arbitrarily defined as regions of >1500 kb) were identified at the 8q21.13, 17p12, 17p11.2, 22q13 and Xq25 chromosome segments (one in each chromosome). Interestingly, each of these larger regions has been previously associated with malignancy and contained genes i) relevant to the metastatic process (i.e.: *TPD52*, *FABP5, MAP2K4, LLGL1, TOP3A, ALDH3A2, UPK3A, FBLN1, TYMP*), ii) associated with intracellular signaling processes (i.e.: *PAG1*, *ELAC2*, *RASD1* and *TNFRSF13B*) and iii) genes involved in the regulation of the cell cycle (i.e.: *FLCN*, *PEMT* and *XIAP*); in turn, three of these large CN regions showing CN losses and one with CN gains contained a total of 8 known microRNAs (Table 3).

**Table 2:** Most frequently detected small regions (<1300 kb) of gain and loss in primary sporadic colorectal tumors genotyped on the Affymetrix 500K SNP array platform (n = 23).

Minimal common altered regions (bp)	Region length (bp)	N. of SNPs	Chromosome band	Event	% of altered cases	Gene list
Chr 1: 26,131,131-26,191,419	60,288	16	1p36.11	Deletion	74	*PAFAH2*
Chr 7: 8,255,230-8,280,496	25,266	10	7p21.3	Gain	74	*ICA1*
Chr 7: 10,461,770-10,486,412	24,642	8	7p21.3	Gain	74	–
Chr 7: 12,514,442-12,576,898	62,456	9	7p21.3	Gain	74	*SCIN*
Chr 8: 32,105,734-32,675,812	570,078	196	8p12	Deletion	70	–
Chr 8: 198,834-392,556	193,722	46	8p23.3	Deletion	70	*FAM87A, FBXO25*
Chr 8: 400,640-539,716	139,076	29	8p23.3	Deletion	70	*C8orf42*
Chr 8: 23,264,737-23,277,681	12,944	8	8p21.3	Deletion	70	–
Chr 8: 86,214,670-86,946,337	731,667	52	8q21.2	Gain	65	*LRRCC1, * ***E2F5*** *, * ***CA13*** *, * ***CA1*** *, CA3, CA2*
Chr 8: 87,377,186-87,789,535	412,349	65	8q21.3	Gain	65	***WWP1*** *, FAM82B, CPNE3, CNGB3*
Chr 8: 88,872,540-89,066,702	194,162	24	8q21.3	Gain	65	*WDR21C*
Chr 8: 91,462,487-91,474,759	12,272	2	8q21.3	Gain	65	–
Chr 8: 91,686,333-91,735,940	49,607	10	8q21.3	Gain	65	*TMEM64*
Chr 8: 94,759,374-95,077,320	317,946	44	8q22.1	Gain	65	*RBM12B, C8orf39, TMEM67, PPM2C*
Chr 8: 95,294,349-95,435,061	140,712	28	8q22.1	Gain	65	*GEM*
Chr 8: 95,593,385-95,776,644	183,259	36	8q22.1	Gain	65	*KIAA1429, RBM35A*
Chr 8: 128,638,191-128,724,583	86,392	25	8q24.21	Gain	65	–
Chr 8: 129,180,096-129,268,067	87,971	43	8q24.21	Gain	65	*MIR1208*
Chr 8: 130,906,244-131,222,249	316,005	35	8q24.21	Gain	65	*FAM49B*
Chr 8: 133,845,345-133,868,639	23,294	9	8q24.22	Gain	65	*PHF20L1*
Chr 8: 133,882,656-133,900,665	18,009	6	8q24.22	Gain	65	–
Chr 8: 135,527,585-135,836,235	308,650	97	8q24.22	Gain	65	*ZFAT*
Chr 8: 136,498,075-136,866,133	368,058	74	8q24.23	Gain	65	***KHDRBS3***
Chr 8: 137,055,200-137,091,177	35,977	12	8q24.23	Gain	65	–
Chr 13: 73,603,130-73,627,939	24,809	10	13q22.1	Gain	78	*KLF12*
Chr 13: 74,972,248-75,117,835	145,587	26	13q22.2	Gain	78	*COMMD6, * ***UCHL3*** *, * ***LMO7***
Chr 13: 75,689,304-75,689,865	561	2	13q22.2	Gain	78	–
Chr 13: 76,352,482-76,366,765	14,283	11	13q22.3	Gain	78	*KCTD12*
Chr 13: 78,098,212-78,143,588	45,376	7	13q31.1	Gain	78	*C13orf7*
Chr 13: 78,805,700-79,077,299	271,599	46	13q31.1	Gain	78	*RBM26, NDFIP2*
Chr 13: 79,621,013-79,845,948	224,935	40	13q31.1	Gain	78	***SPRY2***
Chr 17: 10,693,238-11,021,844	328,606	89	17p13.1	Deletion	78	–
Chr 17: 14,234,746-14,967,525	732,779	214	17p12	Deletion	78	–
Chr 17: 14,984,724-15,082,587	97,863	18	17p12	Deletion	78	*PMP22*
Chr 18: 41,130,655-41,494,986	364,331	134	18q12.3	Deletion	91	*SLC14A2*
Chr 18: 45,410,728-45,497,910	87,182	29	18q21.11	Deletion	91	–
Chr 18: 45,654,114-46,036,475	382,361	144	18q21.11	Deletion	91	*MYO5B, CCDC11*
Chr 18: 46,252,199-46,288,353	36,154	12	18q21.11	Deletion	91	–
Chr 20: 37,766,095-38,339,016	572,921	131	20q12	Gain	83	*HSPEP1*
Chr 20: 51,012,908-51,013,194	286	2	20q13.2	Gain	83	–
Chr 20: 52,991,500-54,234,439	1,242,939	325	20q13.2	Gain	83	*CBLN4*
Chr X: 134,159,698-134,160,254	556	2	Xq26.3	Gain	57	–
Chr X: 151,650,011-151,652,710	2699	2	Xq28	Gain	57	–

Genes which have been associated with cancer are shown in bold.

**Table 3:** Most frequently detected extensively altered chromosome regions with CN changes (>1500 kb) in primary sporadic colorectal tumors genotyped on the Affymetrix 500K SNP array platform (n = 23).

Extensively altered regions (bp)	Region length (bp)	Chromosome band	Event	% of altered cases	Gene list
Chr 8: 80,831,670-82,390,493	1,558,823	8q21.13	Gain	65	*HEY1, MRPS28, * ***TPD52*** *, ZBTB10, ZNF704, * ***PAG1*** *, * ***FABP5***
Chr 17: 11,135,229-14,009,355	2,874,126	17p12	Deletion	78	*DNAH9, ZNF18, * ***MAP2K4*** *, MIR744, MYOCD, * ***ELAC2*** *, HS3ST3A1, MIR548H3, COX10*
Chr 17: 16,270,540-19,616,367	3,345,827	17p11.2	Deletion	78	*TRPV2, C17orf45, C17orf76, ZNF287, ZNF624, CCDC144A,* ***TNFRSF13B*** *, C17orf84, * ***FLCN*** *, COPS3, NT5M, MED9, * ***RASD1*** *, * ***PEMT*** *, RAI1, SREBF1, MIR33B, TOM1L2, LRRC48, ATPAF2, C17orf39, DRG2, MYO15A, ALKBH5, * ***LLGL1*** *, FLII, SMCR7, * ***TOP3A*** *, SMCR8, * ***SHMT1*** *, NOS2B, TBC1D28, TRIM16L, FBXW10, FAM18B, PRPSAP2, SLC5A10, FAM83G, GRAP, EPN2, B9D1, MIR1180, MAPK7, MFAP4, ZNF179, SLC47A1, * ***ALDH3A2*** *, SLC47A2, * ***ALDH3A1*** *, ULK2*
Chr 22: 43,616,234-49,576,671	5,960,437	22q13	Deletion	57	*ARHGAP8, PHF21B, NUP50, C22orf9, MIR1249, * ***UPK3A*** *, FAM118A, SMC1B, RIBC2, * ***FBLN1*** *, ATXN10, * ***WNT7B*** *, C22orf26, MIRLET7A3, MIRLET7B, PPARA, PKDREJ, GTSE1, TRMU, CELSR1, GRAMD4, CERK, TBC1D22A, FAM19A5, C22orf34, BRD1, ZBED4, ALG12, CRELD2, PIM3, IL17REL, TTLL8, MLC1, MOV10L1, PANX2, TRABD, TUBGCP6, HDAC10, MAPK12, MAPK11, PLXNB2, FAM116B, SAPS2, SBF1, ADM2, MIOX, TMEM112B, NCAPH2, SCO2, * ***TYMP*** *, KLHDC7B, CPT1B, CHKB, MAPK8IP2, * ***ARSA*** *,SHANK3, ACR, RABL2B*
Chr X: 120,721,375-126,726,076	6,004,701	Xq25	Gain	57	*GRIA3, THOC2, MIR220A, * ***XIAP*** *, STAG2, SH2D1A, ODZ1, WDR40C, WDR40B, CXorf64*

Genes which have been associated with cancer are shown in bold.

### Chromosomal regions showing high-level CN gains

The highest levels of genetic amplification were detected for the 7p15.2, 8q24.21, 13q12.13 and 20p12.3 chromosome bands with maximum fluorescence intensity log_2_ ratios of 0.99 (0.23±0.11), 1.45 (0.35±0.15), 1.47 (0.31±0.22) and 0.96 (0.28±0.11), respectively (Table 4). Several genes which are potentially involved in the pathogenesis of CRC are localized in these four chromosomal regions. Among others, these include the *CYCS* and *UPP1* genes on chromosome 7p, the *MYC* gene at chromosome 8q24.21, the *HSPH1* and *CDX2* genes at chromosome 13q and the *CDC25B*, *PLCB4*, *TNFRSF6B*, *OGFR*, *NTSR1*, *CDH4*, *CYP24A1* and *RGS19* genes in chromosome 20. The most commonly amplified single region (18/23 cases; 78%) corresponded to a region localized at chromosome 20q11.22 identified by the SNP_A-2220183 and the SNP_A-2039695 at the 33,776,127 bp and 33,954,944 bp positions, respectively (Table S1).

**Table 4:** Most frequently detected high-level amplified chromosome regions (average log_2_ copy number ratio ≥0.22) containing genes commonly associated with cancer in primary sporadic colorectal tumors genotyped on the Affymetrix 500K SNP array platform (n = 23).

Amplified chromosome regions (bp)	Chromosome band	Mean Log_2_ Ratio	Maximum Log_2_ Ratio	% of altered cases	Cancer associated genes
Chr 7: 21,060,948-21,773,238	7p15.3	0.22	0.51	57	*SP4*
Chr 7: 25,072,457-29,780,614	7p15.2	0.23	0.99	52	***CYCS**, CHN2, JAZF1, HOXA1, HOXA4, HOXA5, HOXA7, HOXA9, HOXA10, HOXA11, HNRPA2B1*
Chr 7: 30,433,934-47,043,330	7p15.1	0.24	0.69	52	*SFRP4, AMPH, RALA, INHBA, PPIA, IGFBP3*
Chr 7: 47,249,414-48,538,115	7p12.3	0.23	0.51	57	***UPP1***
Chr 7: 50,305,027-50,512,587	7p12.2	0.24	0.51	61	*DDC, IKZF1*
Chr 8: 128,130,968-129,218,353	8q24.21	0.35	1.45	61	***MYC***
Chr 13: 22,371,210-23,251,245	13q12.12	0.29	0.81	57	*SACS*
Chr 13: 23,722,973-24,224,179	13q12.12	0.30	0.90	57	*ATP12A, PARP4*
Chr 13: 25,516,360-33,070,797	13q12.13	0.31	1.47	61	*BRCA2, RXFP2, HMGB1, * ***HSPH1*** *, SLC7A1, FLT1, FLT3, * ***CDX2*** *, PDX1, GTF3A*
Chr 20: 3,590,646-3,775,309	20p13	0.28	0.62	52	***CDC25B*** *, SIGLEC1, GFRA4*
Chr 20: 6,077,268-10,228,083	20p12.3	0.28	0.96	52	***PLCB4*** *, PLCB1*
Chr 20: 33,776,127-33,954,944	20q11.22	0.27	0.51	78	*RBM39, PHF20*
Chr 20: 47,898,202-49,082,996	20q13.13	0.27	0.55	74	*ADNP, BCAS4, PTPN1, CEBPB, SNAI1*
Chr 20: 52,203,846-52,261,791	20q13.2	0.27	0.55	74	***CYP24A1***
Chr 20: 59,237,873-59,740,719	20q13.33	0.27	0.59	74	***CDH4***
Chr 20: 59,926,031-62,297,793	20q13.33	0.28	0.82	74	*TAF4, SS18L1, LAMA5, * ***GATA5*** *, SLCO4A1, * ***NTSR1*** *, * ***OGFR*** *, * ***TCFL5*** *, DIDO1, BIRC7, EEF1A2, PTK6, STMN3, * ***NFRSF6B*** *, TPD52L2, SOX18, * ***RGS19*** *, OPRL1,*

Genes which have been commonly associated with colorectal cancer are shown in bold.

Only those regions with recurrently amplified DNA copy-number found in at least half of the cases, are listed.

Interestingly, we recorded a statistically significant association between tumour grade and presence of gains/amplifications at the 20p13 chromosomal region localized between the 2,574,587 and 2,993,797 bp positions and assessed by 66 SNPs with a greater frequency of well- vs moderately-differentiated tumours- (11/13 (85%) vs 2/8 (25%); p = 0.005) among cases with this chromosomal alteration.

### Recurrent chromosomal breakpoints identified by SNP-arrays

Based on the analysis of the distribution of chromosomal breakpoints defined by the SNP-arrays, four recurrent chromosomal breakpoints (arbitrarily defined as DNA segments showing CN changes in more than one third of the cases) were identified at chromosomes 1p12, 8p12, 17p11.2 and 20p12.1 (Figure S1). Chromosomes 1, 8 and 20 showed a high number (>145) of different breakpoint regions with a variable and heterogeneous distribution; in contrast, a highly prevalent breakpoint region was identified in the centromeric portion of chromosome 17p, between the genome coordinates 20,156,497 bp and 22,975,771 bp (15/19 patients with abnormalities for this chromosome), and a minimum size of 28.2 Mb for the recurrent breakpoint. In these 15 cases, the first gene affected on the retained telomeric side of the breakpoint region was the *CYTSB* gene and the first constantly deleted gene on the centromeric side was the *FAM27L* gene. Interestingly, in 13 of these 15 patients a preferential breakpoint occurred at the 21,769,828–22,975,771 genome coordinate where the *FAM27L* gene is coded.

### Correlation between the chromosomal changes detected by SNP-arrays and both iFISH and RQ-PCR studies

In order to evaluate the consistency of the chromosomal changes identified by the SNP-arrays, iFISH analysis were performed in parallel for a total of 24 chromosome regions from 20 different chromosomes. Overall our results showed a high degree of correlation (mean r^2^ of 0.73±02; range: 0.65 to 0.91) between both methods, including when such analysis was restricted to the most frequently altered regions (r^2^≥0.67) (Table 5).

**Table 5:** Primary colorectal cancer with liver metastasis (n = 23): correlation between the numerical changes detected by each individual iFISH probe used and the CN changes identified for the corresponding single nucleotide polymorphisms (SNPs) through SNP array studies.

Chromosomal region identified by the iFISH probe	R^2^/*P-*value
1p36	0.75/<0.001
1q25	0.75/<0.001
2p24	0.65/0.001
3q26	0.81/<0.001
5p15.2	0.65/0.001
6q23	0.67/<0.001
7q31	0.67/<0.001
8p22	0.81/<0.001
8q24	0.79/<0.001
9p21	0.91/<0.001
9q34	0.77/<0.001
10q23	0.68/<0.001
11q22	0.82/<0.001
12p13	0.76/<0.001
13q14	0.74/<0.001
13q34	0.78/<0.001
14q32	0.82/<0.001
15q22	0.72/<0.001
17p13	0.80/<0.001
18q21	0.75/<0.001
19q13	0.65/<0.001
20q13.2	0.80/<0.001
21q22	0.74/<0.001
22q11.2	0.83/<0.001

R^2^: Coefficient of correlation.

**Figure 3:**
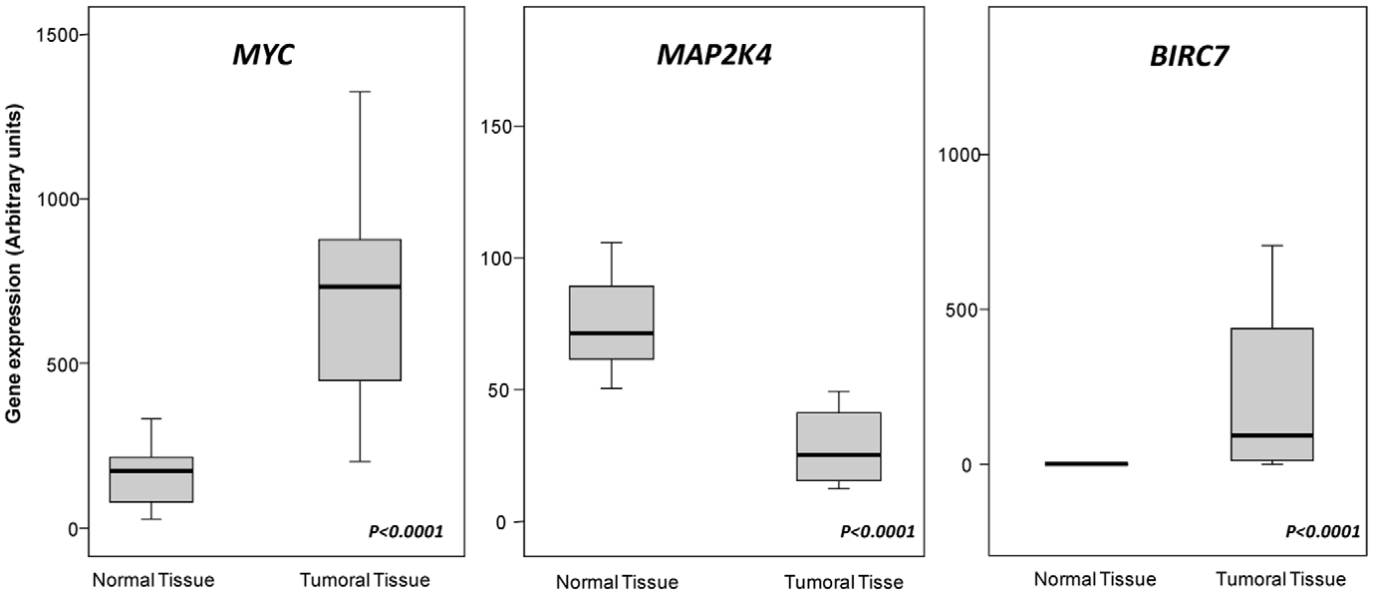
Expression levels of MYC, MAP4K and BIRC7 mRNA as assessed by RQ-PCR in metastatic CRC tumors and their corresponding paired normal tissue (n = 18). Note that MYC and BIRC7 mRNA levels from metastatic CRC tumours samples are significantly higher than in their paired normal tissues (p<0.0001). By contrast, MAP4K mRNA levels in metastatic CRC tumors are significantly lower than normal (p<0.0001).

In order to assess the impact of the information generated by SNP arrays, the expression of three genes (*MAP2K4*, *MYC* and *BIRC7*) was further analyzed in detail using RQ-PCR. As expected from the SNP-array data, the *MYC* and *BIRC7* relative transcript levels were up-regulated in 15/18 (83%) and 14/18 (78%) tumours analyzed, respectively. Conversely, the *MAP2K4* gene was down-regulated in 16/18 (89%) tumours (Figure 3). Upon comparing the results obtained with the two methods, a significant (p<0.001) correlation was observed between the microarray data and the expression of the three genes evaluated by RQ-PCR techniques with correlation coefficients (r^2^) of 0.88, 0.66 and 0.64 for *MAP2K4, MYC* and *BIRC7* genes, respectively.

## Discussion

In this study we describe a comprehensive map of the genetic abnormalities present in primary tumors from metastatic CRC through the usage of high-resolution 500K SNP arrays. To our knowledge this is the most extensive study using high-resolution SNP-arrays to define the genetic alterations in this subgroup of CRC patients. Overall, our results confirm previous analyses using chromosome banding techniques [20], CGH [5], SKY [21], aCGH [6], [10] and low-resolution 50k SNP-arrays [22].

Previous reports in which similar SNP-array tools have been applied to investigate the genetic profile of non-metastatic CRC [23] have shown in a subset of patients with advanced carcinomas in the absence of liver metastases (n = 18), a relatively low frequency of 1p, 8p, 9q, 14 and 17p losses and unique amplifications at chromosome 20q. Interestingly, among our series of metastatic CRC patients the frequency of losses at the same chromosomal regions was strikingly higher: 1p, 74% vs 11%; 8p, 78% vs 33%; 9q, 35% vs 6%; 14, 65% vs 39%; and; 17p, 83% vs 33%. In turn, we also detected additional amplifications at 7p, 8q and 13q, as well as at the 20q chromosomal region. In line with our observations, Al-Mulla *et al* [24] also found that, once compared to patients without metastatic disease (n = 30) CRC patients with liver metastases (n = 26) more frequently displayed losses of chromosomes 1p, 4, 5q, 8p, 9p, and 14q. Altogether, those results indicate that the genetic profile of metastatic CRC is defined by imbalanced gains/amplifications of chromosomes 7p, 8q, 13q and 20q together with losses of the 1p, 8p, 9p, 14q and 17p chromosomal regions [5], [20], [25]–[27]. In addition, here we describe new recurrently altered regions that contain cancer genes, many of which have been previously involved in the pathogenesis of CRC, at the same time, we provide detailed characterization of recurrent chromosomal breakpoints most frequently occurring in primary tumours from CRC patients who had developed liver metastases.

Interestingly, a relatively high degree of correlation was found between the cytogenetic alterations detected by SNP-arrays and iFISH studies. Despite this, slight differences were noted between both techniques. On one hand, these were due to the lower sensitivity of the SNP-array vs. iFISH for the identification of chromosomal abnormalities present in only a small proportion of all cells in the sample (i.e. secondary genetic lesions absent in the ancestral tumour cell clones) [28]. On the other hand, they were attributable to the increased sensitivity of the SNP-array vs. iFISH studies as regards identification of small interstitial changes [11]. In this regard, our results show occurrence of a high number of CN changes involving minimal/small regions (<1.3 Mb) and to a less extent, also extensive/large (>1.5 Mb) regions which frequently went undetectable by iFISH. Interestingly, several of these small and large altered regions contain cancer-associated genes known to be involved in CRC and/or the metastatic process: i.e. the *TPD52* [29], *FABP5* [30], *MAP2K4* [31], *LLGL1* [32], *FBLN1* [33] and *TYMP* [34] genes.

Among all human chromosomes, chromosomes 17 and 18 were those more frequently found to be altered in our series, their abnormalities typically consisting on extensive deletions involving the *TP53* and *DCC* genes, respectively, in addition to other tumor suppressor genes, such as *MAP2K4* at 17p12. A potential role for chromosome 18q in the development of CRC with associated liver metastases has been previously reported [35]; in this regard, decreased expression of Smad4 in addition to *DCC*, has been pointed out as a potential target protein coded in chromosome 18q since it is associated with both liver and lymph node metastases [36]. In line with these findings we also identified loss of the *SMAD4* gene in the great majority (83%) of the metastatic cases analyzed. By contrast, the most frequently (78% of cases) amplified region was found in chromosome 20, at 20q11.22. This is a relatively small region of 178,817 bp which harbors 8 known genes, half of which have been associated with CRC: *TNFRSF6B* [37], *OGFR* [38], *NTSR1* [39] and *CDH4* [40]. Among these genes, overexpression of *TNFRSF6B* -a gene that belongs to the tumor necrosis factor receptor (*TNFR*) super-family- has been reported in advanced stages of CRC [37] and other tumors of the gastrointestinal tract [41], in association with an increased resistance to adjuvant chemotherapy [42]; in turn, increased *NTSR1* expression has been reported as an early event in colon tumorigenesis that contributes to tumor progression and an aggressive clinical behavior [39]. Similarly, we also identified amplification and overexpression of the *MYC* gene at 8q24 in the great majority of the primary tumors, which have both been previously suggested to be involved in disease progression to a metastatic tumour [28]; [43].

From the clinical point of view, gain/amplification of 20p13 was associated with a higher frequency of well vs. moderately-differentiated tumours. Noteworthy, this chromosomal region contains genes which have been previously associated with disease progression. Accordingly, Miyoshi N *et al* have recently suggested that overexpression of the *TGM2* gene in CRC patients is associated with a shorter overall survival [44] and expression of the *PTPRA* gene has been recurrently associated with progression of gastric cancer, including lymphovascular invasion and liver/peritoneal dissemination [45], [46].

Apart from defining the most frequently altered genes in metastatic CRC, this study was also aimed at detailed characterization of the most frequent recurrent breakpoint regions associated with such genetic changes. The number of different breakpoints detected within individual chromosomes is usually considered as a surrogate marker for chromosomal instability in cancer. In the present study, we found 245 different breakpoints for chromosome 1. This frequency is significantly higher than that reported by others using aCGH analyses of CRC without distant metastases: 16 different chromosomes breakpoints found, in a group of 32 patients [10]. These results suggest that advanced-stage and metastatic CRC could be associated with a greater number of breakpoints and higher chromosomal instability. In line with this hypothesis, Knutsen *et al* [21] found 407 chromosomal breakpoints in 15 CRC cell lines, using spectral karyotyping with a high frequency of recurrent breakpoints in the centromeric (p11 to q11) or pericentromeric (p11.2 and q11.2) regions of chromosomes 12, 13, 14, 15, 17 18 and 20. Interestingly, in this latter study Knutsen *et al* [21] also found recurrent breakpoints at 17p11.2 in 6/15 cell lines.

In the present study, a high percentage of cases showed recurrent breakpoints for chromosomes 1, 8, 17 and 20. Most interestingly, breakpoints at chromosome 17p were preferentially localized at the genome coordinate 20,156,497–22,975,771 bp at 17p12 (15/23 cases); in most of these cases (12/15 cases), the breakpoint was restricted to the genome coordinate (21,769,828–22,975,771 bp) which maps for the *FAM27L* gene, a gene whose function remains to be elucidated. Whether, disruption of the *FAM27L* gene may also play a role in the malignant transformation and/or the metastatic process of CRC into the liver in addition to, inactivation of *TP53* and inhibition of apoptosis [47], [48], remains to be elucidated. Nevertheless, it should be noted that Camps *et al* [10] have shown a higher frequency of 17p11.2 breakpoints in CRC patients with positive (8/16) vs. negative (4/16) lymph nodes using aCGH. This breakpoint has been previously associated with an homogeneous genetic profile defined by a higher frequency of abnormalities of chromosomes 1p, 7, 8, 13q, 18q and 20q and an adverse clinical outcome [35], [49]–[52]. Other recurrent chromosomal breakpoints found in our patients were localized in the 1p12, 8p12 and 20p12.1 chromosomal regions. Previous studies suggest that genes typically deregulated by these chromosome breaks included the *REG4* [53] and *NOTCH2* [54] genes at chromosome 1p12, *EIF4EBP1* [55] and *FGFR* [56] at chromosome 8p12, and the *FOXA2* [57] gene at chromosome 20p12; all these genes have been associated with the development and progression of CRC and the metastatic process in a variety of human cancers, including the development of liver metastases in CRC [53]–[57]. Additional GEP and functional studies as well as direct comparison of paired primary and metastatic tumours are required to validate our findings and to gain further insight into their role in metastatic CRC patients.

## Supporting Information

Figure S1 Primary colorectal cancer with paired liver metastasis (n = 23): Identification of recurrent chromosomal breakpoint regions for the 1p12, 8p12, 17p11.2 and 20p12.1 chromosome regions as defined by the Affymetrix 500K SNP array genotyping platform. Breakpoints occurred in 9 cases (39%) at the 118097448-120939802 genome coordinate for chromosome 1 (panel A), in 8 cases (35%) at the 37770635-38405382 coordinate for chromosome 8 (panel B), in 15 cases (65%) at the 20156497-22975771 position for chromosome 17 (panel C) and in 9 cases (39%) at the 14921777- 16089156 genome coordinate for chromosome 20 (panel D).
(4.70 MB TIF)

Table S1 Most frequently detected amplified regions (for >3 contiguous SNPs with average log2 copy number ratio >0.22) in primary colorectal tumours from metastatic CRC patients genotyped on the Affymetrix 500K SNP array platform (n = 23). Only recurrently amplified DNA copy-number regions found in at least half of the cases, are listed.
(0.10 MB DOC)
