# Supplementary figures and images for: Mapping of Genetic Abnormalities of Primary Tumours from Metastatic CRC by High-Resolution SNP Arrays
Source: PLoS One. 2010 Oct 29;5(10):e13752. doi: 10.1371/journal.pone.0013752 (PMC2966422; doi:10.1371/journal.pone.0013752)

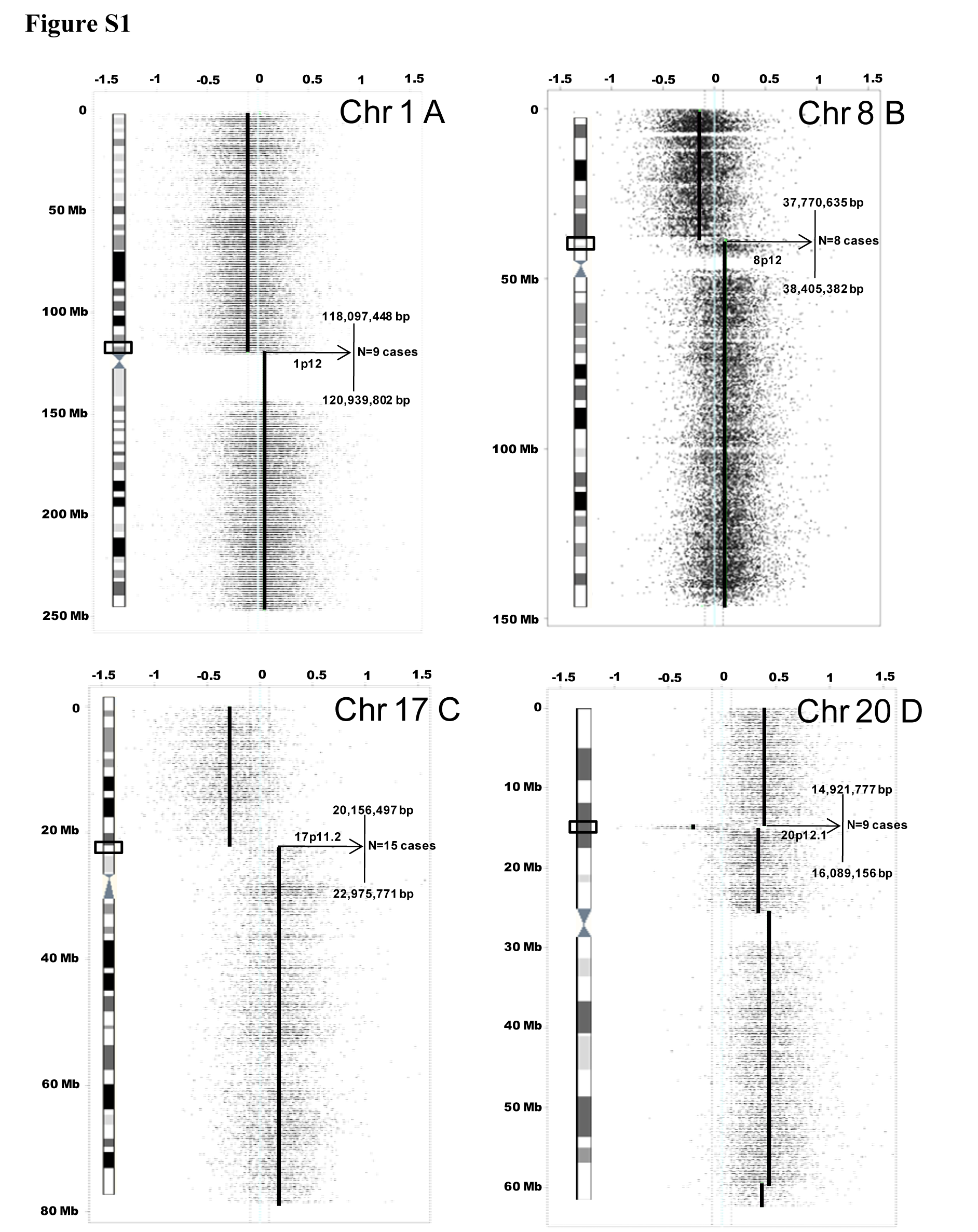

Supplement: Figure S1 — Primary colorectal cancer with paired liver metastasis (n = 23): Identification of recurrent chromosomal breakpoint regions for the 1p12, 8p12, 17p11.2 and 20p12.1 chromosome regions as defined by the Affymetrix 500K SNP array genotyping platform. Breakpoints occurred in 9 cases (39%) at the 118097448-120939802 genome coordinate for chromosome 1 (panel A), in 8 cases (35%) at the 37770635-38405382 coordinate for chromosome 8 (panel B), in 15 cases (65%) at the 20156497-22975771 position for chromosome 17 (panel C) and in 9 cases (39%) at the 14921777- 16089156 genome coordinate for chromosome 20 (panel D). (4.70 MB TIF) [file pone.0013752.s001.tif]
