# Supplementary material for: Mapping of Genetic Abnormalities of Primary Tumours from Metastatic CRC by High-Resolution SNP Arrays
Source: PLoS One. 2010 Oct 29;5(10):e13752. doi: 10.1371/journal.pone.0013752 (PMC2966422; doi:10.1371/journal.pone.0013752)
Supplement: Table S1 — Most frequently detected amplified regions (for >3 contiguous SNPs with average log2 copy number ratio >0.22) in primary colorectal tumours from metastatic CRC patients genotyped on the Affymetrix 500K SNP array platform (n = 23). Only recurrently amplified DNA copy-number regions found in at least half of the cases, are listed. (0.10 MB DOC) [file pone.0013752.s002.doc]

**Table S1.**

| **Amplified chromosome**  **regions (bp)** | **Chromosome**  **band** | **Mean Log_2_ Ratio** | **Maximum Log_2_ Ratio** | **% of altered cases** |
| --- | --- | --- | --- | --- |
| Chr 7: 256,369-294,586 | 7p22.3 | 0.23 | 0.61 | 57 |
| Chr 7: 3,296,034-3,316,151 | 7p22.2 | 0.25 | 0.59 | 61 |
| Chr 7: 3,397,398-3,488,329 | 7p22.2 | 0.22 | 0.51 | 57 |
| Chr 7: 5,342,068-5,563,375 | 7p22.1 | 0.23 | 0.60 | 65 |
| Chr 7: 6,483,965-6,673,156 | 7p22.1 | 0.22 | 0.68 | 65 |
| Chr 7: 8,255,230-8,280,496 | 7p21.3 | 0.22 | 0.51 | 74 |
| Chr 7: 11,707,008-11,716,404 | 7p21.3 | 0.25 | 0.99 | 70 |
| Chr 7: 20,340,816-20,346,394 | 7p21.1 | 0.22 | 0.51 | 65 |
| Chr 7: 21,060,948-21,773,238 | 7p15.3 | 0.22 | 0.51 | 57 |
| Chr 7: 24,144,398-24,239,684 | 7p15.3 | 0.22 | 0.51 | 52 |
| Chr 7: 24,322,185-24,416,579 | 7p15.3 | 0.22 | 0.51 | 52 |
| Chr 7: 24,978,567-24,990,225 | 7p15.3 | 0.22 | 0.51 | 52 |
| Chr 7: 25,072,457-29,780,614 | 7p15.2 | 0.23 | 0.99 | 52 |
| Chr 7: 30,433,934-47,043,330 | 7p15.1 | 0.24 | 0.69 | 52 |
| Chr 7: 47,249,414-48,538,115 | 7p12.3 | 0.23 | 0.51 | 57 |
| Chr 7: 50,305,027-50,512,587 | 7p12.2 | 0.24 | 0.51 | 61 |
| Chr 7: 54,095,049-54,233,081 | 7p11.2 | 0.22 | 0.49 | 61 |
| Chr 7: 57,480,106-57,494,714 | 7p11.2 | 0.23 | 0.60 | 65 |
| Chr 8: 91,462,487-91,474,759 | 8q21.3 | 0.37 | 2.16 | 65 |
| Chr 8: 128,130,968-129,218,353 | 8q24.21 | 0.35 | 1.45 | 61 |
| Chr 8: 129,268,681-130,294,656 | 8q24.21 | 0.35 | 1.45 | 61 |
| Chr 13: 18,671,379-18,807,765 | 13q12.11 | 0.29 | 0.81 | 52 |
| Chr 13: 21,633,957-21,655,355 | 13q12.11 | 0.29 | 0.81 | 52 |
| Chr 13: 21,767,986-22,101,448 | 13q12.11 | 0.29 | 0.81 | 52 |
| Chr 13: 22,371,210-23,251,245 | 13q12.12 | 0.29 | 0.81 | 57 |
| Chr 13: 23,722,973-24,224,179 | 13q12.13 | 0.30 | 0.90 | 57 |
| Chr 13: 24,515,343-25,503,127 | 13q12.13 | 0.30 | 2.61 | 57 |
| Chr 13: 25,516,360-33,070,797 | 13q12.13 | 0.31 | 1.47 | 61 |
| Chr 13: 34,181,075-34,241,426 | 13q13.2 | 0.33 | 1.31 | 61 |
| Chr 13: 94,150,135-94,170,855 | 13q31.3 | 0.30 | 0.79 | 70 |
| Chr 13: 99,316,581-99,515,767 | 13q32.3 | 0.29 | 0.61 | 65 |
| Chr 13: 102,033,267-102,045,693 | 13q33.1 | 0.30 | 0.61 | 70 |
| Chr 20: 174,08-172,128 | 20p13 | 0.27 | 0.47 | 52 |
| Chr 20: 3,590,646-3,775,309 | 20p13 | 0.28 | 0.62 | 52 |
| Chr 20: 5,964,236-5,997,522 | 20p12.3 | 0.29 | 0.63 | 61 |
| Chr 20: 6,077,268-10,228,083 | 20p12.3 | 0.28 | 0.96 | 52 |
| Chr 20: 20,094,851-20,100,855 | 20p11.33 | 0.27 | 0.66 | 52 |
| Chr 20: 29,480,104-29,526,117 | 20q11.21 | 0.32 | 1.50 | 74 |
| Chr 20: 33,776,127-33,954,944 | 20q11.22 | 0.27 | 0.51 | 78 |
| Chr 20: 43,331,553-43,528,383 | 20q13.12 | 0.27 | 0.58 | 74 |
| Chr 20: 46,875,410-46,881,367 | 20q13.13 | 0.30 | 1.13 | 74 |
| Chr 20: 47,898,202-49,082,996 | 20q13.13 | 0.27 | 0.55 | 74 |
| Chr 20: 52,644,833-52,735,018 | 20q13.2 | 0.27 | 0.46 | 74 |
| Chr 20: 52,737,405-52,737,405 | 20q13.2 | 0.28 | 0.46 | 65 |
| Chr 20: 59,237,873-59,740,719 | 20q13.33 | 0.27 | 0.59 | 74 |
| Chr 20: 59,926,031-62,297,793 | 20q13.33 | 0.28 | 0.82 | 74 |
| Chr X: 125,114,477-125,146,027 | Xq25 | 0.29 | 0.78 | 57 |
| Chr X: 126,529,787-126,726,076 | Xq25 | 0.41 | 1.77 | 57 |
| Chr X: 128,458,733-136,800,659 | Xq25 | 0.27 | 2.60 | 52 |
| Chr X: 148,331,391-154,569,169 | Xq28 | 0.27 | 2.12 | 52 |
